# "The Hospital" Nursing Mirror

**Published:** 1897-12-04

**Authors:** 


### "The Hospital" Nursing Mirror.

Being the Nursing Section of "The Hospital."

[Contributions for this Section of "The Hospital" should be addressed to the Editor, The Hospital, 28 & 29, Southampton Street, Strand London, W.C., and should have the word "Nursing" plainly written in left-hand top corner of the envelope.]

### News from the Nursing World.

### A COMMEMORATION GIFT.

Her Majesty has been pleased to hand the sum of £1.100, the gift presented by the deputation received at Windsor on November 19th from the Girls' Friendly Society, to the hon. treasurer of the Queen's Commemoration Fund (Mr. Sydney Holland) on behalf of the Queen Victoria Jubilee Institute for Nurses.

### THE QUEEN AND THE QUEEN'S NURSES.

Amongst the patients of the Queen's Nurses at Tunbridge Wells was a poor old dame. She was so overcome with gratitude for their kindness and attention that she firmly resolved that Her Majesty should know of all their goodness to her, and she meant to write and tell her so herself as soon as ever she got well. The old lady died, but before she passed away she obtained the promise of a lady visitor to carry out her wish. In due course Lieut.-Colonel Sir Fleetwood Edwards replied, by command of Her Majesty, thanking the lady for her letter, and informing her that "Her Majesty was particularly interested in learning the contents of her letter, and to have received the touching message from the poor woman who expressed her great gratitude for the comfort and attention afforded her by the attendance of the Jubilee nurses during her fatal illness."

### BROMPTON JEWISH CEMETERY.

The news that the Jewish Cemetery at Brompton is to be put in order is very welcome, and especially gratifying to The Hospital, seeing that the movement originated in a paragraph that appeared in The Hospital "Mirror" some weeks ago. We drew attention to the melancholy state of this burial ground, and our remarks reached the eye not only of the Jewish authorities, but also of a gentleman whose parents lie buried there. This gentleman has announced his intention of defraying all expenses. It will be a gain to the immediate neighbourhood, for though nothing will be permitted to infringe upon the sacred character of the ground, yet a well-kept, quiet spot is very grateful in the midst of so much traffic. The fact that it is sacred will ensure its quietude. Otherwise it would be invaded by many whose idea of pleasure is to test the strength of their lungs shouting at one another.

### GRATEFUL MAIDSTONE.

The grateful citizens of Maidstone have raised a special subscription amongst themselves, with the kind intention of bestowing a souvenir of the town on every nurse employed during the typhoid epidemic. Pretty silver medals have been designed for this purpose, and will be formally presented on December 8th, when the Lord Mayor of London will be the guest of the Mayor of Maidstone. A large number of nurses are still in the town, but their work is decreasing, and their services will probably be dispensed with at no distant date. Private persons, who desire that the nurse who has attended them during the epidemic shall receive the same token of recognition, may purchase a medal and present it to her.

### FIGHTING TYPHOID AT LYNN.

The example set by the Princess of Wales has been followed by some of her loyal Norfolk neighbours, who have sent useful garments for the invalids at King's Lynn. By means of the subscriptions collected by the ladies of the town, all the present wants of the sick are supplied from day to day. Clothing is distributed from a small central depôt managed by ladies, and the Mayor has arranged elsewhere for giving out food and other relief. A diet-kitchen provides the necessary meals for convalescents, and beef tea for patients, who are nursed in their homes. It is said that there was an extraordinary demand for night-gowns and shirts on the morning following the announcement of the Princess's gifts to the town. Doubtless each poor inhabitant felt that her sick son or daughter must get well if she could secure a garment sent direct from the Royal home at Sandringham.

### CITY OF LONDON UNION INFIRMARY.

Pending the completion of a new nurses' home, the Infirmary Committee have directed arrangements to be made for housing the nurses and probationers. The Guardians have sanctioned the expenditure of not more than £100 for furnishing the nurses' home and temporary wards.

### LOVE INTERVENES.

Lady Griselda Ogilvie, daughter of the Dowager Countess of Airlie, is about to renounce the profession of nursing, of which she is a fully-trained adept, and to enter the bonds of matrimony. She is much attached to her hospital work in Newcastle, which she was about to resume when these new interests prevented her doing so.

### GLOSSOP GOSSIPS.

Miss Ellen Mary Warrener, for some time matron of Wood's Hospital, Glossop, is a plucky woman, and in a case of uncommon difficulty has acted with resolution and prudence. A number of charges of cruelty were made against her, with the result that she was unceremoniously dismissed from her post. But they vanished like snow in a thaw when brought into a court of law. Alderman John Barnes, Mayor of Glossop, it was stated, had acted in the matter from a full conviction that the charges were true. On learning that they were not he was prepared to apologise and pay whatever damages were awarded. Miss Warrener, however, cared little for damages, and willingly accepted £10 10s. We must congratulate Miss Warrener on having come out of the affair with the sympathy of judge, jury, and public.

### AXMINSTER CARNIVAL.

The support of the cottage hospital at Axminster culminates in an annual *fête*. The town is illuminated, and a grand procession parades the streets before throngs of admiring spectators. Many interesting tableaux were arranged this year, six of which received prizes, We are sorry to note that a hospital ward was again one of them; a representation of doctor, nurse, patient, and mother turned a solemnity into a spectacle. The idea strikes false on that innate sense of propriety which is always felt, even if it be not expressed.

### LOWESTOFT PROVIDENT NURSES' ASSOCIATION.

Two things attractive of interest transpired at the annual meeting of the Lowestoft Provident Nurses' Association. The one, that the directors recognise the educational value of the nurse, and secondly, that it is a more or less successful attempt to teach the poor to provide against sickness. Of course, the backbone of the association is the subscriptions of the generous, which have been to a great extent assured by the indomitable pluck and hard work of the Secretary, Miss F. Cockburn; but five nurses can no longer supply the demands for their services, and the income fell just a little short of the expenditure. The district nurse receives from her patient one penny per visit, or per day if more than one visit is needed. The maternity cases, which keep four nurses fully employed, pay either 2s. or 3s. subscription annually, according to the earnings of the applicant, and 2s. or 4s. a week when the nurse attends. The nurse in these cases goes into residence and superintends the household as well as the patient. Visitors amongst the poor may have a nurse for their sick by paying fees on the same scale. The accounts are carefully kept and audited, but it can hardly be properly callsd a provident association if the payment is not made before hand.

### LICHFIELD DOCTORS AND THE NURSING HOME.

Mr. Seckham has offered a site whereon to build a nursing home for Lichfield. The medical men of the town have inspected it, and found that it is in many ways unsuitable for the purpose as a permanent home; they have, therefore, counselled the committee to ask Mr. Seckham for a larger site (which he has intimated his willingness to give), higher on Beacon Hill. For some reason the committee has declined the amendment by 14 votes to 9, and the doctors have published their reasons for proposing it. The committee has certainly shown the courage of its opinions in going against the unanimou decision of seven medical men, but are they wise?

### SUNBEAM LEAGUE, CARDIFF.

Mrs. Franklen Evans, in her speech to the members of the Infirmary Guild, referred to the effort now being made in Cardiff to induce children to help children. The district has been divided into wards, and when the children of a ward collect or save £50 a cot is named after it in the infirmary. The Park Ward has already its Sunbeam League cot. Much depends upon the way a thing of this kind is managed before anything in praise or condemnation can be said. If personal interest is awakened in the child's heart towards the little sufferer in the cot, and it willingly exercises self-denial in order to contribute its mite to assuage the want of another, well and good; the seed is of the right kind, and the harvest blest. But if, on the other hand, pressure is put on the child's generosity, if it be permitted to beg indiscriminately, the tares will undoubtedly appear, possibly in the shape of a grudge against the charity, possibly by the breakdown of self-respect which begging often entails.

### WHITCHURCH.

The Whitchurch (Salop) Cottage Hospital has just completed the eleventh year of its existence. The income is the highest but one of any previous year, notwithstanding a special appeal for the enlargement of the hospital as a memorial to the Queen's Diamond Jubilee reign, towards which about £1,000 was subscribed in less than three weeks. The average stay in hospital of the 86 patients was 26¼ days, who, with the staff, were maintained at a daily cost of 9¼d. each inmate, a result due to the excellent management of the Matron, Miss Griffiths, supplemented by various gifts in kind. The dispensary and the district and maternity nursing branches fully maintain their usefulness. In the former 149 tickets were issued, while in the latter 1,262 visits were paid, of which 874 were free; 563 dressings were performed, and 490 free materials supplied. Deducting the cost of the outdoor work, the average cost of each in-patient is about 3s. 4½d. per day including establishment charges. Daily average of patients, 6¼d.

### DUNDEE SICK POOR NURSING SOCIETY.

A large and influential assembly gathered to the annual meeting of the Dundee Sick Poor Nursing Society. Eight years ago two nurses were appointed, and now the staff consists of a superintendent and nine nurses. Miss Mackay, the superintendent, has been awarded the silver medal by the London Council, which was presented to her a short time ago by the Princess Louise. The result of the meeting shows that more liberal support is needed by the committee to meet the continually increasing demand for nurses; that the evils of the present condition of midwifery practice urge immediate reform; and that the practical help given by the poor to the poor was much in excess of what is imagined. Altogether the society seems in a thoroughly healthy state; the work it does is well done in such a way as not only to obtain recognition at headquarters, but to win the approval and gratitude of the sick. It proclaims itself ready to tackle with resolution one of the greatest wrongs done to women and children, and exhibits a wise recognition of the principle of mutual help which it is the glory of the true district nurse to teach and to use for the advantage of the community.

### SHORT ITEMS.

The newly instituted district nursing association of New Malden and Coombe is to be known as the Victoria Nursing Association.—The contract for building the nursing home for Godalming has been given to Messrs. Mitchell Bros. The price is £878.—A number of lady nurses left England on Thursday, the 25th inst., to nurse the Plague in Bombay.—The Harborne Nursing Society is flourishing. The nurse made on an average ten visits a day. The balance in hand is £38, and that of the Samaritan Fund £25.—The proceeds of Mr. D. S. Salmond's lecture on South Africa in the Town Hall, Wooler, have been devoted to the fund for the nurse lately established in the district.—Brooks, the man who is accused of having defrauded Nurse Trucler of about £25 by representing to her that he was engaging her for a patient, and then making off with her luggage, seems from the police reports to have been implicated in about a dozen other similar cases.—Miss Lillian Tanner, of the Taunton and Somerset Hospital, sailed on November 25th, in the *Himalaya*, for India, to nurse the plague.

### Practical Aspects of a Nurse's Life.

By Sister Grace.

### XI.—STAFF NURSES.

When probationers have completed their training and prefer hospital to private work, they naturally hope to be made staff or night nurses, and the most promising are usually chosen to fill these posts, both of which have heavy responsibility attached to them.

To my mind the position of a staff nurse is one of considerable difficulty. Much devolves on her; she is in a position of command with servants, patients, and nurses, without the full authority of a sister. Especially with the probationers I have seen difficulties arise; the fault probably is not on one side only, but I would beg all probationers to remember that the position of their staff nurse is far from easy, and to try, by their loyal readiness to carry out her wishes, to lighten her daily work. And to the staff nurse herself may I offer this advice in all friendliness. Remember that you have but recently been on exactly the same footing as the nurses who are now under your orders; do not afford them a chance (as I once heard) to make unpleasant allusions to an uncivil but well-known proverb that begins, "Put a beggar on horseback," &c. When first promoted it requires tact and judgment to manage your new work without giving offence. There are those who will quarrel with you however much you strive to work with justice and kindliness, but see that they do not do so without real reason.

Whilst speaking of the social difficulties of the staff nurse, I should like to warn you against the pernicious habit of making violent friendships. With probationers it is to be deplored, but as you ascend in the nursing scale it is still more dangerous. From our life being spent chiefly in one groove it usually happens that we have not a very wide range of subjects for conversation, therefore if friends are together on every available opportunity, it is to be expected that very purposeless talk becomes the rule. The subjects under discussion must frequently be the patients, the nurses, the sisters, and the doctors, and this soon degenerates into petty gossip, not always of a strictly harmless nature. Therefore, avoid these close intimacies, which after all are more suitable for schoolgirls than for women with a serious work in life.

### Importance of the Post in Surgical Wards.

The staff nurse is a valuable person in all wards, but I think her importance is most felt in surgical work. She must in all respects take the place of the sister in her absence, and in surgical and accident work especially that means that she must be ready for all emergencies; serious accidents must often be immediately prepared for the theatre, therefore it is imperatively necessary that she should know all the dressings that are generally employed in all operations. This knowledge does not come in a month, nor in many months; it requires close attention and hard study for several years to make a really competent surgical nurse, who shall not be embarrassed by any difficulty that may arise, however suddenly and inopportunely.

You must take every opportunity of noticing the preparations that are made for the different operations, and the different arrangements and dressings preferred by the several surgeons.

The longer I am in hospital life, the more I am convinced that a nurse's knowledge of, and proficiency in, her profession, depends more upon her own powers of observation and private study than upon the amount of instruction imparted to her by others, though it is very pleasant to obtain this latter help, and encourages one much, but no amount of instruction from others will take the place of a ready and acute observation on your own part. Sometimes I am inclined to think that things are made almost too easy nowadays, and that the thorny path of past days, when we had to "pick up" the precious pearls of knowledge, sometimes under great difficulties, had its advantages.

### The Duty of Instructing Probationers must fall to a certain degree on the staff nurse. Custom varies; some sisters who are very energetic and painstaking try to carry out this duty thoroughly themselves, others pass it by almost entirely. In any case it is certain that, whether compulsory or not, the staff nurse has many opportunities of helping those who work under and with her. Endeavour to induce in them habits of method and order, and it is evident that you cannot do this without being yourself methodical and orderly. It is very hard on probationers to be constantly called away from some appointed task to help in work which should have been done before, or to clear away unsightly *débris* which by a little care and forethought need not have accumulated. Remember, too, not to exact too much waiting on. I think probationers are almost always ready to give cheerful service to a woman they can respect and look up to, but they resent too many airs of superiority.

Finally, by your own demeanour and circumspect conduct under all circumstances endeavour to keep up a good tone among the nurses. Your position with regard to the sister is that of a valued lieutenant to his captain, and everyone knows how the manner and habits of the sister give the tone to the whole ward; you, therefore, as her faithful lieutenant, must help to carry out her principles, and remember how all those younger than yourself in position will carefully note all your words and actions.

Loyalty and strict obedience to the sister are duties that I hope it is unnecessary to insist on; indeed, I think it but seldom that staff nurses fail in this respect. It is manifest that without these qualities life in the wards between the sister and staff nurse would be a continual strife, but when I speak of loyalty I mean much more than a careful carrying out of orders. I mean that you should endeavour in all your work to do everything exactly as she would wish it done, and to see that trifling matters of routine, perhaps of no real importance, are arranged in strict accordance with her wishes. Unity of purpose between your sister and yourself makes the work of a ward go very smoothly. I feel pleasure in recording here how many pleasant hours in the past, and pleasant recollections in the present, I owe to the loyal devotion, never-failing willingness, and untiring zeal of almost all those who acted as my staff nurses.

### The Royal British Nurses' Association.

A pleasant afternoon was spent by nurses and their friends on Monday, when an "At Home" was held at the offices of the Association. Music, recitations, and tea were all most attractive, and, no doubt, will cause a still larger gathering to assemble on December 22nd, the next guest afternoon. The *Nurses' Journal*, we learn, is to become a monthly publication from January next. We congratulate the Editorial Committee upon the excellent tone which pervades the last quarterly number. It augurs well for the new venture.

### Post-Graduate Clinics for Nurses.

By a Trained Nurse.

XXXVII.—THE NURSING OF TYPHOID (*continued*).

I hope nurses in charge of cases of typhoid will give a trial to the soft pads I described in a previous paper rather than bed-pans, which when diarrhœa is present lead to so much disturbance of the patients. The burning of these pads is a boon to the community, and if such a system were universal the outbreak of "secondary" cases, so common after a typhoid epidemic, would be minimised to vanishing point. If the nurse, however, elects to employ the traditional bed-pan, let her carefully warm this, and oil the edges before passing it under the patient. After use let her carefully sponge the parts with warm water—if sore and irritable through constant wetness she should use barley-water—dry with a soft towel, and dust with a bismuth powder. "Secondary" epidemics arise through the lack of time in disinfecting the excreta and a hasty emptying into the drainage system. The nurse, in her anxiety to get rid of the excreta, does not give time for the stools to become impregnated with the disinfectant. She empties the whole away before the destructive solution has had time to destroy the germ; the rush of water used for flushing so dilutes the disinfecting medium that the germs lodge undestroyed in the sewers. The stools should be saturated with strong carbolic acid, and be allowed to stand a few minutes before being emptied into the drains.

Diet.

After reminding the nurse that otorrhœa is a by no means uncommon accompaniment of typhoid fever, and that in such a case the ears should be periodically syringed with warm water, with the addition of Sanitas or boracic lotion and boracic dusted freely in after the syringing, I will go on to the important question of feeding the patient. A typhoid case must perforce live on liquids for some four or five weeks, and much ingenuity will be needed to guard against the monotony entailed by this somewhat restricted dietary. The rooted repugnance shown by typhoids to the conventional milk and soda or barley water is very intelligible. For such a dietary is not only most unpalatable, but its monotony constitues a mild cruelty of the sick room. Where so many delicious combinations of milk diet exist, no valid excuse exists for the eternal "feeder" of milk and soda.

The dieting of typhoid varies according to the constipated or loose condition of the bowels. If constipation be present, beef teas and meaty infusions may be beneficial. If diarrhœa be a marked symptom, these must be restricted or withheld. Despite their fever and thirstiness, most typhoids complain with reason of the enormous quantities of fluid given them. The condition of their digestion frequently makes it necessary to so highly dilute the milk given, that in order to get enough nourishment huge quantities must be taken. When diarrhœa is present the large amount of fluid taken adds considerably to the looseness and frequency of the motions, so that it is well to know how the food may be concentrated so as to counteract this, remembering that such concentration must be consistent with digestibility.

In every type of typhoid I have found ice-cream a most valuable, digestible form of nourishment, readily taken by young and old. Its consistence partly satisfies the craving for solids, added to which a large amount of easily-digested nourishment is afforded in small bulk. If the ice cream can be prepared at home, so much the better. If not, it should be obtained from a reliable source, made of "double-cream," and only slight flavourings of vanilla, coffee, or chocolate allowed. Fruit flavourings are inadmissible. Unquestionably the tympanites or bowel distension, so painful a feature of some typhoidal cases, is much increased by large quantities of fluid. I have found in such cases the utmost good result from the use of ice-cream, and some other concentrated foods to be hereafter mentioned.

All nurses are familiar with instances of child-typhoids absolutely refusing all milk foods, and having to be fed nasally. Few children over five will refuse ice-cream.

Valentine's meat juice may be safely given to typhoids suffering from extreme diarrhœa, as this has not the effect of fresh infusions in increasing looseness of the bowels. This meat juice makes an excellent meat jelly when added to isinglass dissolved in warm water, put on ice, and given in cold spoonsful. This valuable meat juice should never be mixed with acids or spirits, and few patients like it when given pure or in warm water. Added either to aerated or barley water, mixed in a little iced champagne, sherry, or tea, or given with crushed ice, it is most palatable and nourishing. In cases of extreme exhaustion it may be given hypodermcally or added to nutrient enemata, and is excellent with chicken or mutton broths.

I have never met with a typhoid patient—however antipathetic these were to milk—who did not enjoy koumiss made at home in the following way.

Take a dessert-spoonful of fresh yeast which has been washed several times in water till no bitter taste remains, add this to a quart of fresh cow's milk sweetened to taste with sugar of milk, and at blood-heat temperature. Put this in patent-stoppered bottles if preferred, but it occurred to me once to unstopper a syphon, put the milk in this, and refasten the stopper. I found the syphons so much more convenient that I have always used them since. But whether bottles or syphons be chosen fill them and put them over night (twelve hours) in a warm place such as you would choose for bread to rise in. The temperature necessary is from 67 to 70 deg.

Next morning you will find a frothy, delicious milk compound which, when given cold, has never in my experience been rejected by the most irritable stomach. It is frequently digested even when peptonised milk does not agree. And one of its chiefest benefits is that treated thus the milk may be given pure. It is, therefore, a valuable concentrated form of nourishment, which even in small quantities will sustain a patient's strength. For typhoids pour the koumiss several times from one tumbler to another to expel the air.

The great point in feeding sick people is never to give the same food twice consecutively. Suppose a typhoid to take nourishment every two hours, what a simple matter it is to give him something novel at each feeding time. Beginning at eight a.m., he might take coffee-flavoured milk. At ten o'clock a small quantity of peptonised cocoa. By midday he will appreciate something more solid, and will enjoy some Valentine's meat juice jelly, or frozen beef essence. At two p.m. some ice-cream will ring the changes, while tea-flavoured milk at four p.m. will bring back a reminiscence of afternoon tea. At six o'clock some chicken broth, followed at eight p..m by cream and whisky or cream and champagne. Koumiss may be given at ten p.m. At midnight veal tea or light broth, followed at two a.m. by the whipped yolk of an egg with brandy. The next meal, at four a.m., might consist of whey with a little cream in it; while at six a.m. a dessert spoonful of malt extract dissolved previously in a small quantity of warm water and mixed with milk might be given.

### In Memoriam.

WALTER HAYES BURNS.

Lives of great men all remind us
We may make our lives sublime,
And, departing, leave behind us
Footprints in the sands of time.

Since 1725 St. George's, Hanover Square, has been consecrated to the worship of God. It is a structure resembling as much as possible the Quaker's meeting house, yet possessing a solid dignity of its own. Below the high stained glass windows at the east end is a picture of the Last Supper, in a frame square, heavily gilt, the dim light falls softly on its rich, harmonious colouring, and gleams as it touches the golden moulding that surrounds it.

On November 27th, at noon, the chancel was filled with flowers, the air laden with their perfume. Wreaths and crosses of every conceivable shape and size were piled against all and every projection. The floor, excepting only a pathway in the middle, was carpetted with them, and still attendants came with more until there was no place for them. Conspicuous on the left, raised against the chancel stalls, was a magnificent Maltese cross. The arms were solid with Neapolitan violets and fringed with lilies of the valley and their young yellow green leaves. The centre was thick with the same lilies and the rare and delicate Cattleya orchid. The card upon it bore testimony that this was the nurses' tribute to their chairman from the members of the Royal National Pension Fund for Nurses. A beautiful floral tribute came also from the secretary and staff of the same fund.

Presently the hurrying to and fro ceased, a clergyman took his place and read the usual morning prayer. The spectators found seats, and two mourners came and knelt throughout the service. A few minutes after its conclusion the minute bell began to toll and soft music came from the organ. At twenty-five minutes past the hour the clergy and choir passed slowly to meet the funeral train. Then all was silent until a voice was heard proclaiming the centre truth of revelation, "I am the resurrection and the life, saith the Lord; he that believeth in me, though he were dead, yet shall he live;" and all that was of earth of a noble man was borne shoulder high amid thronging friends heavy laden with sorrow to its resting-place in front of the chancel steps. The appointed Psalms and "Lead, kindly light, amid the encircling gloom" (A. and M. 266) were sung by the choir, and the beautiful service for the burial of the dead proceeded in a quiet, broken occasionally by sobs. Another hymn (437), an echo of the triumphant chant of victory, "For all the saints who from their labours rest," was then sung, and followed by the Dead March in "Saul," and the mourners went forth to commit the body of their beloved to the grave. To have been present at such a ceremony was a privilege and an inspiration. Is it not ever a privilege to render honour to whom honour is due? Those for whom the late Mr. W. H. Burns has built a refuge against sickness will hold his memory in grateful remembrance. It is also an inspiration. Longfellow has crystallised the thought into words for us. We, though we may not hope to leave the "footprints in the sands of time," can take courage from a noble life nobly lived, and largely spent for the benefit of others less fortunate than himself.

### Wants and Workers.

Nurse Lilly would be glad to hear of a nurse to share her home for the sake of companionship. Furnished bedroom 3s. 6d. weekly, without attendance. Ivy Cottage, Bunbury Heath, near Tarporley, Cheshire.

### The Kodak Exhibition.

The New Gallery, Regent Street.

The most wonderful thing about the many wonderful things at this exhibition is the manner in which a new enterprise grows. The Kodak is no longer a toy, an amusement for the passing hour, it is a practical invention of manifold utility, and affords work to some thousands of workmen. New York is the headquarters of Messrs. Eastman, but the English branch is assuming large proportions, and the factory at Harrow-on-the-Hill already employs over a couple of hundred men. The Kodak enters very slightly into the surgery and laboratory, but the films, papers, and other preparations for X ray work are quite specialities of the firm. A new folding camera at £2 2s. is so very neat, complete, and handy that it is certain to be a favourite. To be able to take a dozen snapshots with an instrument no bigger than a medium-sized pocket-book is delightful.

### Where to Go.

Sir Squire Bancroft has kindly consented to give his popular reading of Charles Dickens' "Christmas Carol" on Wednesday evening, December 8th, at Surbiton Assembly Rooms, in aid of the funds of the national refuges and training ships *Arethusa* and *Chichester*, a society which during the past 54 years has received over 14,000 poor boys and girls. Commander Hugh H. Hannay, R.N., will preside at the forthcoming reading, and a large number of influential residents at Surbiton have booked seats for the occasion.

The Working Ladies' Guild.—The winter sale of the Working Ladies' Guild will be held at Windsor House, Mount Street, on December 8th, 9th, and 10th. Open from twelve to six. Her Royal Highness the Duchess of Connaught will attend the sale. Admission—first day, 2s. 6d.; second and third days, 1s.

Distressed Gentlewomen's Aid Association.—A musical entertainment and tableaux vivants will be given at St. Martin's Town Hall, Trafalgar Square, on December 3rd, at eight p.m., in aid of the above. Tickets 4s., 2s. 6d., and 1s.

### Appointments.

MATRONS.

West Ham Workhouse Infirmary.—Mrs. C. Okell enters upon the duties of Matron at the above institution at Christmas. She was trained at St. Bartholomew's, and has been assistant matron at St. Saviour's Infirmary, Dulwich, and matron of the Bridgewater Infirmary, Somerset.

### Minor Appointments.

The Princess Mary Village Homes, Addlestone, Surrey.—Miss Henrietta Hamilton, formerly Sister Sophia of the London Hospital, and afterwards matron of the Princess Alice's Memorial Hospital, Eastbourne, and the Lady Strangford Hospital, Port Said, has been appointed Superintendent of these homes.

Cardiff Union Workhouse.—The new trained nurse for this workhouse is Miss Ellen Connelly, who was trained at St. Olave's Union Infirmary, Rotherhithe, and who was later staff nurse under the St. Olave's Board of Guardians.

### The Book World for Women and Nurses.

SICK-ROOM COOKERY AND HOSPITAL DIET, WITH SPECIAL RECEIPTS FOR CONVALESCENT AND DIABETIC PATIENTS. By MAUDE EARLE. With Notes on the Feeding of Infants by FRANK C. MADDEN, F.R.C.S. (London: Spottiswoode and Co. 1897. Price 3s. 6d.)

This ought to be a good book, written as it is by a lady who is a staff teacher of the National Training School of Cookery and a lecturer on sick-room cookery at the London Hospital, the Brompton Hospital for Consumption, &c., and after carefully reading it we are able to say that it is a most useful and reliable guide to the art of which it treats. A short sketch is first given of the classification and the food values of the various articles of diet. This will no doubt be found of interest by general readers, although to modern nurses who have attended lectures on elementary physiology it may seem like going over old ground. The diet scales which are arranged for various diseases demand a word of caution, for useful as they are likely to be to those who recognise the danger of a little knowledge, they certainly require to be read with judgment. It must be understood that they do but indicate the kinds of diet which are commonly found useful in these several diseases, and the things that may and may not be safely suggested in the absence of instructions by the doctor. Before the recipes begin there is a short explanation of cookery terms which might with advantage have been extended. Some very judicious remarks are then made about the making of beef-tea, and various recipes are given, the intention of which is to produce a tea containing the coagulable albumen. It is quite recognised by the author that this is not the most tasty form of beef-tea that can be made, but it is properly urged that it is by far the most nutritious, "which is everything when the case of the patient is serious." We may, perhaps, point out that nurses are so accustomed to the use of the thermometer and that the exact temperature at which this kind of beef-tea is made is so important, that it might be well to direct that a thermometer should be used and that the temperature should be kept within a certain range. Bad cooks boil their meat, good cooks "simmer" it, and the writer of this book is evidently one of the good ones, but the time is coming when good cooks will do better still and cook things at definite temperatures.

Some very pretty little recipes are given for the cooking of fish, one of the most important articles of sick-room cookery, because it so often constitutes one of the first tentative steps towards convalescence. We quite agree that steaming fish is the lightest and most digestible way of serving it. For those who have advanced a little in their recovery, "Sole à la Colbert" is a very dainty little dish, while for those who like tasty things the recipe for "Sole au gratin," with its chopped mushrooms, its shred of onion, and its surrounding of tomato sauce, is very appetising. A whole series of dishes is given by which to pacify the cravings of the diabetic, and tempt him not to transgress the hard rules laid down for the regulation of his diet; and then, curiously enough, there is a chapter on poultices!—a distinctly unappetising excursion into foreign topics. This, surely, is a chapter gone astray. We must not omit to give a word of praise to Mr. Frank Madden's interesting little article on "The Preparation of Food for the Bottle-fed Infant," a subject on which, as the medical superintendent of the Hospital for Sick Children, Great Ormond Street, he speaks with authority. Altogether, this is a very interesting book, and certainly will be useful. The patients who are fed according to its teaching will undoubtedly live well, and if the recipes are carefully followed their digestion need not suffer. In acute cases it is no part of a nurse's duty to cook; she is too busy with other things. Even in such cases, however, it is an immense advantage that she should know how things ought to be done, and be able to recognise when they are done wrongly where the error lies. But in the nursing of chronics and convalescents a nurse may make herself invaluable and simply indispensable by her ability to tempt appetite by dainty dishes.

BURDETT'S OFFICIAL NURSING DIRECTORY, 1898. Compiled and Edited, with the assistance of a Small Committee of Medical Men and Matrons, by SIR HENRY BURDETT, K.C.B. (London: The Scientific Press. Price 5s.)

This handsome volume, full as it is of useful information, is clearly but an earnest of what it is fated to become in a few years' time, when both nurses and the public shall have experienced the advantage of being able to see at a glance the status, the training, and the experience of the nurses whose qualifications are given in its pages. The difficulties in the way of compiling such a directory must have been very considerable, and in fact it is stated in the preface how great have been the obstacles which have had to be overcome. Nursing is at present an ill-defined calling, ungoverned, some perhaps would say untrammelled, by any legal restrictions. No definition has yet been arrived at which will, without doing injustice, meet all cases, and will in each instance answer the question—a nurse or not a nurse? The compilation of such a list as this must thus involve the exercise of a wise and just discretion, absolutely untainted by any bias in favour of this, that, or the other institution, and we cannot but look upon it as a fortunate thing for nurses that this work has been undertaken by one who has not only in many ways shown his sympathy with nursing in its every phase, taking the word in its widest meaning, but has for so long been favourably known for his skill and impartiality in the superintendence of what one may call "Directory" work. The first half of the volume is occupied by a mass of useful information, which we should think nurses would find it very difficult to obtain in one volume anywhere else;—an outline of the principal laws affecting nurses; a list of hospitals and poor-law infirmaries which train probationers; lists of nursing institutions, separating those managed by a committee from private agencies and homes; a list of institutions where nurses are employed but not trained; a sketch of various foreign and colonial hospitals and nursing institutions; and lists of training schools for attendants on the insane, and of various other institutions. Perhaps we are wrong in speaking of these pages as being occupied by "lists"; for, in fact, what is given under each head is something much more than the word list expresses. We find, for example, under the several training institutions the number of beds, the name of the matron, the number and classification of the nurses, and all sorts of details as to the training given and the fees required. If even as a mere handbook for those entering upon nursing work we fancy this Directory will have a large popularity. To those, however, who are already nurses, and also to the public, who are naturally anxious for some sort of security as to the training and experience of those to whom they trust so much, the Directory proper will be the main point of interest. In regard to this, we may at once say that it does not as yet appear to cover the entire field, and that as things stand the absence of a nurse's name from the list cannot be taken as anything against her—except, perhaps, as showing a little pig-headedness in not sending her name in; but so far as it goes the work is one of great value, and evidence is given on every page of the labour which has been bestowed upon it. "What the Directory does is to state the facts as nearly as they can be ascertained. Anyone possessing this Directory can ascertain the experience or training of each nurse whose name appears in it." So says the preface, and so seems to be the fact. Name, address, present position, where trained and when, what appointments have been held and dates of the same, what certificates are held, such as the L.O.S., and what association, such as the R.B.N.A., she is a member of. To the public and the medical profession the book will no doubt be a work of much utility, while to each nurse whose name appears in it it will not only be a useful work of reference, but a handsomely bound certificate.

### The Camberwell Scandal.

THERE is evidently something behind the smoking scandal at Camberwell. It will probably not be divulged, and yet, unless it be, the action of the Guardians in regard to the accused nurses must seem arbitrary and incomprehensible. The naked facts, stripped of the graceful but imaginary dressing by the press, are that the night superintendent and four of her subordinates were called before the Guardians and asked if they smoked. On replying in the affirmative they were asked to send in their resignations, which they did. Naturally the accused were indignant, and they urged as an excuse that they only smoked in their own rooms, in consequence of the stench arising in their quarters' situated in the old workhouse not long since condemned as unsuitable for the patients and medical men to inhabit; but the Guardians refused to hear any defence. Without either agreeing or disagreeing with the action of the Guardians, we may say that so far, they were within their rights. If they choose to dismiss from their service all who smoke they can do so, although, in ordinary fairness, they should state this condition at the time of engaging those whom they employ. Where the Guardians did a wrong and an injustice to these nurses was in letting it go forth that the charge brought against them was one of "unseemly conduct," which might mean anything, and practically carried with it a deprivation of all future employment. There was no evidence, so far as we can discover, of any "unseemly conduct" such as has been alleged, for smoking in private, however imprudent and undesirable it may be for women is not a crime in the opinion of the majority. The action of the Guardians is supposed by some to have originated in the malicious representations of a nurse; but this is merely a guess, and seems inadequate to account for it, especially as one of the persons implicated in the charge of smoking is the night superintendent, who is responsible to the medical officer for the night nursing of the infirmary, and who holds therefore a position of authority.

Taking the other side of the dispute; the argument for the defence that the stench in their quarters was only endurable by the help of a cigarette seems more or less of an excuse, the truth appearing to be that the accused (and others also) liked smoking, and saw no harm in enjoying it in their own rooms in the absence of any definite commands on the subject. The accounts of the smells and rats seem greatly exaggerated. A number of the nurses, enjoying good health, declare that they have smelled no smells and seen no rats, and at the time of our visit neither were apparent, nor were there any disinfectants about, as there would probably have been in the event of much unpleasantness. The timber in the flooring and wainscote was also sound, the one nibble taken by a rodent being in the night superintendent's room, which we did not see as she was asleep. Still, in view of the building having been "condemned," we think the Guardians ought to make sure about the drains before dealing in so high-handed a manner with those who differ from them on the question of smoking.

The infirmary is well nursed, the majority of the nurses being popular and efficient. The patients are excellently looked after, the food and cooking are good. But undoubtedly there is much that might be improved in the appointments of the infirmary. For instance, even the new apartments provided for the nurses have only one bath attached, and that is not a fixture. The cubicles are small and divided only by curtains, and one lavatory arrangement has no direct communication with the outer air; but the fact remains that for the most part the nurses are satisfied, and the majority of those in the condemned structure have no desire to be moved. Nevertheless, in face of the condemnation of the workhouse as a dwelling by medical authority, it is not right to keep them there, and the sooner the new home, for which negotiations have been going on since the spring, is ready the better.

We are glad to hear that the Guardians have reconsidered their decision, and have unanimously granted the accused nurses their testimonials. The charge of "unseemly conduct" was a serious one to bring against nurses in their employ. It covers so wide a field that the imprudence of smoking, to which they confess, was trifling in comparison, and, if persisted in, must to a great extent have ruined their career. Only moral delinquency merits so severe a punishment, and even then justice is sometimes tempered with mercy.

### Everybody's Opinion.

[Correspondence on all subjects is invited, but we cannot in any way be responsible for the opinions expressed by our correspondents. No communication can be entertained if the name and address of the correspondent is not given, as a guarantee of good faith but not necessarily for publication, or unless one side of the paper only is written on.]

### HOUSEKEEPING.

MISS MENA BIELBY, 115, Marylebone Road, N.W., writes: If your correspondent "A Constant Reader," inquiring in THE HOSPITAL of November 13th as to housekeeping on £40 per year, likes to write to me, I shall be very pleased to help her in the matter, as I have for years lived in London, in a flat, on a far smaller sum, and have learnt by experience how to reduce housekeeping expenses and trouble to a minimum.

"BRAES O' MAR" writes: Will you kindly oblige by inserting the following remarks in answer to "H. B." and "A Constant Reader," apropos of their letters in THE HOSPITAL of November 13th. I did not venture last week to give my opinion to "H. B." and "A Constant Reader" as I thought it probable that some one with more experience or a readier pen would be able to give advice. I may say at once that I consider "H. B." is making a great mistake in working for such a small salary. I shall perhaps be accused of being mercenary, but I am nothing of the kind; it is simply a question of common sense and reasonable looking ahead a little. Your food at 7s. a week will come to £18 a year. Then allow 1s. a week for what you call housekeeping expenses. That will, with £5 for your laundry (which you do not mention), bring it up to £25 10s. or thereabouts. This leaves you the handsome (?) salary of £16 a year with which to provide clothes and travelling expenses, not to mention such things as charity, luxuries, or saving anything for illness and old age. No district nursing association should offer, and no district nurse with proper training should undertake, any charge under £80 a year inclusive. It is only just to her patients and to herself to ask it, to the former because without good food, a good holiday, and an easy mind how can she do her patients the most good possible? And just to herself because the labourer is worthy of his hire, and after all her hard work in hospital surely she deserves better than £16 a year. As to "A Constant Reader," her monetary matters are not so bad, and I have no doubt that living on £40 a year can be done with proper management, but what cannot be done is that a nurse can work twelve hours on her district a day and attempt housekeeping besides. One wonders in what corner of the United Kingdom an association exists as to expect such a thing of any one weak woman! I have been a district nurse for a number of years, and have many friends also district nurses, and I know from experience (personal and otherwise) that eight hours a day is as much as anyone is fit for, although, of course, if occasion demands, one would not hesitate to work over hours. I am sorry I can give no hints as to the right spending of "A Constant Reader's" salary, as I have (since working single-handed) always lived in lodgings. I hope I am not encroaching too much on valuable space, but there seems so much to say on this subject that one hardly knows when to stop.

### For Reading to the Sick.

THE SEASON OF ADVENT.

"KNOWING the time, that now it is high time to awake out of s'eep: for now is our salvation nearer than when we believed."—*Romans* xiii. 2.

**Verses.**

Hark, a thrilling voice is sounding,
Christ is nigh it seems to say;
Cast away the works of darkness,
O ye children of the day.

Wakened by the solemn warning,
Let the earth-bound soul arise;
Christ, her Sun, all ill dispelling,
Shines upon the morning skies. —*E. Caswall.*

Christ, Whose glory fills the skies,
Christ the true, the only Light,
Sun of Righteousness, arise,
Triumph o'er the shades of night;
Dayspring from on high be near;
Daystar, in my heart, appear.

Dark and cheerless is the morn
Unaccompanied y Thee,
Joyless is the day's return
Till Thy mercy's beams I see,
Till Thy inward light impart,
Glad my eyes, and warm my heart.
—*Charles Wesley.*

'Tis but a little while
And He shall come again,
Who died that we might live, Who lives
That we with Him may reign.
Then, oh, my Lord prepare
My soul for that great day,
Oh, wash me in Thy precious blood
And take my sins away. —*Dr. Bonar.*

**Reading.**

"Behold, I stand at the door and knock." Give admittance unto Christ, and deny entrance to all others. He will be thy faithful and provident Helper in all things, so as thou shall not need to trust in men. For men will change and quickly fail; but Christ remaineth for ever and standeth by us firmly unto the end.—*Thomas à Kempis.*

Do you know the feeling of expecting a friend to come and he delays? Do you know what it is to be in anxiety lest something should happen which may happen and may not, or to be in suspense about some important event, which makes your heart beat when you are reminded of it, and of which you think the first thing in the morning? Do you know what it is so to live upon a person who is present with you that your eyes follow his, that you read his soul, that you see all the changes in his countenance, that you anticipate his wishes, that you smile in his smile, and are sad in his sadness, and are downcast when he is vexed, and rejoice in his successes? To watch for Christ is a feeling such as all these, as far as feelings of this world are fit to shadow out those of another.—*Dr. Newman.*

### Novelties for Nurses.

WE have received a specimen of Dr. Thomalla's hygienic underclothing. The material is an all-wool closely and evenly-woven woollen texture, singularly free from the irritating quality frequently associated with woollen garments. Such well-woven a fabric could not fail to be thoroughly durable, and the garment we have before us is very carefully and neatly finished. The seams are secured by an effective herring-bone stitching, the fronts are bound with a strong silk binding, and the trimming is in neat woollen lace.

### Notes and Queries.

**The contents of the Editor's Letter-box have now reached such unwieldy proportions that it has become necessary to establish a hard and fast rule regarding Answers to Correspondents. In future, all questions requiring replies will continue to be answered in this column without any fee. If an answer is required by letter, a fee of half-a-crown must be enclosed with the note containing the enquiry. We are always pleased to help our numerous correspondents to the fullest extent, and we can trust them to sympathise in the overwhelming amount of writing which makes the new rules a necessity. Every communication must be accompanied by the writer's name and address, otherwise it will receive no attention.**

*Probationer's Examinations.*

(76) Would you kindly let me know if reading, writing, and arithmetic would be the only examinations in which a probationer would require to pass, or are they only given as a test before entering the hospital?—*Nellie Boyd.*

Reading, writing, and arithmetic form the entrance examination for a probationer. Later, as she is trained, she has special examinations on the work she has learnt.

*How to Train for a District Nurse.*

(77) Will you kindly tell me how to train for a district nurse, cost, &c.? I have been a year in a London Hospital. Also if all district nurses have to undertake midwifery cases without a doctor?—*L. M. E.*

The necessary qualifications are given in "Burdett's Official Nursing Directory" as follows: (*a*) Training at some approved general hospital or infirmary for not less than one year; (*b*) approved training in district nursing for not less than six months, including the nursing of mothers and infants after childbirth; (*c*) nurses in country districts must have had at least three months' approved training in midwifery. The Queen's Nurses are carefully chosen, and trained especially for district work.

*Probationers.*

(78 (1) Will you kindly tell me if I could enter any "large or small" hospital as a probationer free? I have had no experience whatever. (2) Is 24 years about the age to enter as probationer at any hospital, and should I have any difficulty in obtaining a post as such? (3) Would any infirmary in the suburbs be as well as a general hospital?—*E. B.*

(1) There is a demand for suitable candidates as probationers, and if such you will probably be accepted. (2) 24 is the most suitable age. (3) Choose a good general hospital. See the "Mirror" for April 3rd, 1897. It will help you.

*Convalescent Home.*

(79) Some months ago there was a notice in THE HOSPITAL of a convalescent home founded by a gentleman in memory of his daughter where the beds were free I believe it was for nurses and governesses. Kindly tell me particulars.—*E. Y. Sparrow.*

The matron's address is Miss Longbottom, The Convalescent Home, Castle Hill, Bletchingley, Surrey. The home is in memory of Mr. Partridge's daughter, and is intended for nurses, governesses, clerks, &c.

*"The Hospital" as a Gift.*

(80) Would any nurse or institution like to have a number of copies of THE HOSPITAL and "Mirror" for the past few years, which can be had free if carriage is paid?—*H. and S.*

*Best Course of Training.*

( ) Will you be so kind as to inform me what is the best possible course of nursing training obtainable in this country? May I ask if you would advise attendance at lectures?

A three years' course of training at one of the general hospitals. From your letter we imagine King's College Hospital would suit you. The lectures are frequent, and the nursing well organised. With regard to the question of payment, write direct to the matron.

*Association of Asylum Workers.*

(82) I should be greatly obliged to the editor of THE HOSPITAL if he could give me more particulars of the Association of Asylum Workers.—*Miss Adams.*

Dr. Shuttleworth, Ancaster House, Richmond, Surrey, is the secretary, to whom applications for particulars ought to be made.

*A Male Nurse.*

(83) I know a young man who has just completed his course of training as a male nurse in the Belle Vue Hospital, New York, and has received his certificate there. He is now wishing to obtain work in London, if there is a chance of a good opening for him. Can you give him advice how to proceed, or would he find a better opening in Paris?—*Sister Eleanor.*

There is plenty of work for him in England. He might apply to the Hamilton Association for Providing Male Nurses, 57, Park Street, Grosvenor Square, W. Nurses on the staff receive the fees they earn after a commission is deducted.

*L.O.S.*

(84) Will you kindly tell me if I could enter any examination for L.O.S. certificate. Am assistant nurse in a workhouse infirmary, and have had some experience in midwifery, or should I have to enter a maternity hospital? (2) Would it be any use for me to try a postal course of lectures?—*Nurse F.*

Ask your medical officer if he could and would sign your certificate for practical work; if so, you can study the theory by correspondence. Why not join the Midwives' Institute, 12, Buckingham Street, Strand. You will then have practical advice upon every point.

*Trained Nurses' Annuity Fund.*

(85) Will you kindly inform me where to apply for an annuity from the Trained Nurses' Annuity Fund?—*A. Young.*

R. Gofton-Salmond, Esq., 72, Cheapside, E.C.

